# Bacterial Endocarditis of Anatomically Normal Native Pulmonic Valve with No Predisposing Risk Factors: Case Report and Review

**DOI:** 10.1155/2020/1453126

**Published:** 2020-04-08

**Authors:** Muhannad Antoun, John Deel, Dermot Halpin, Fadi Al-Akhrass

**Affiliations:** ^1^Infectious Disease Department, Pikeville Medical Center, Pikeville, KY, USA; ^2^Cardiothoracic Surgery Department, Pikeville Medical Center, Pikeville, KY, USA

## Abstract

Right-sided native valve infective endocarditis (IE) includes tricuspid valve (TV) and pulmonic valve (PV). It represents 10% of all cases. However, it is more common in persons who inject drugs or in presence of cardiac implantable electronic device (CIED). Pulmonic valve endocarditis is a rare infection and represents ∼1% of all cases. Our case represents a patient with large pulmonic valve vegetation with no known common risk factors for right-sided IE.

## 1. Introduction

Endocarditis is an infection of the endocardium, first described by William Osler in 1885, and most commonly is a bacterial infection. Pulmonic valve endocarditis is a rare condition among all infective endocarditis and usually occurs secondary to valve anomaly, devices placement, or in people who inject drugs (PWID). MRSA is still the most common pathogen causing endocarditis in all valvular structural type.

## 2. Case

A 56-year-old man with history of type 2-diabetes mellitus presented to our facility with few days' history of subjective fever, body aches, nonproductive cough, and left-sided pleuritic chest pain that increases with deep inspiration. The patient is known to have recurrent infected skin cyst with methicillin-resistant *Staphylococcus aureus* (MRSA). The cyst is located over the thoracic back, which required incision and drainage in the past. A few years prior to his current presentation, a vascular surgery with stent placement was performed on the right upper extremity due to vascular stenosis.

Physical exam upon arrival revealed no heart murmur. Lungs auscultation; coarse breath sounds with no wheezing. Abdomen without tenderness or distention. Lower extremities with no erythema or ulcerations; pulse is palpable in all four extremities. An infected skin cyst lesion was found over the thoracic back which was lanced at bed side. A very thick purulence was exposed from the cyst. A specimen was sent to the microbiology laboratory for culture, which grew MRSA sensitive to sulfamethoxazole/trimethoprim, clindamycin, tetracycline, and vancomycin with minimum inhibitory concentration (MIC) ∼1  mg/ml. It was intermediate to levofloxacin.

Vital signs were blood pressure 147/85, temperature 98.7 Fahrenheit (F), heart rate of 97 per minute, and respiratory rate 18 per minute. The laboratory findings were as the follows: white blood cells count 17  K/UL, creatinine 0.7  mg/dl, sodium 128  mg/dl, and platelet count 205.

**Figure 1:**
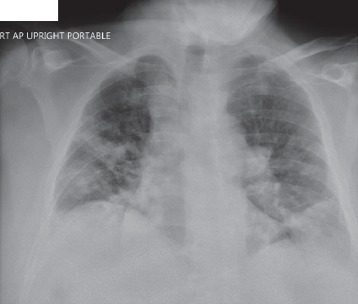
Simple chest XR.

Radiographic study with chest X-ray (Figure 1) showed development of multifocal regions of nodular appearing infiltration within both lungs and possible small amount of bilateral pleural fluid.

**Figure 2:**
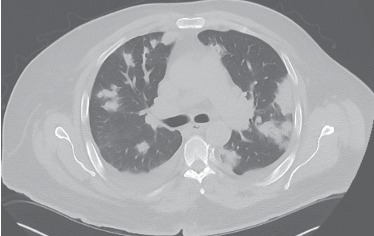
CT scan of the chest without contrast.

Computed tomography (CT) scan (Figure 2) angiogram of the chest was performed, and it showed patchy lung infiltration in both sides compatible with pneumonia. Also, there was mediastinal and bilateral hilar lymphadenopathy.

Blood culture was collected and sent to microbiology which resulted as positive with Gram-positive cocci and that grew MRSA which was sensitive to vancomycin with MIC ∼1  mg/ml, sulfamethoxazole/trimethoprim, clindamycin, tetracycline, and intermediate to levofloxacin.

The patient was initially started on intravenous (IV) vancomycin, IV ceftriaxone, and IV azithromycin, and then the antibiotic adjusted to only IV vancomycin, after obtaining the blood culture result. A transthoracic echo showed no valvular vegetation. The patient continued to be bacteremic with MRSA despite being on antibiotic/vancomycin. The antibiotic was exchanged to IV daptomycin and IV linezolid and IV ceftaroline.

Extensive workup was done to determine the hematogenous focus of infection which is seeding the pathogen into the blood stream. Lumber spine magnetic resonance imaging (MRI) showed no diskitis, osteomyelitis, or epidural abscess. Upper and lower extremities Doppler ultrasound showed no deep venous thrombosis or any arterial stenosis in all 4 extremities. CT scan of abdomen and pelvis with and without contrast did not reveal any intra-abdominal source of infection. Transesophegal echo (TEE) was performed. However, no valvular vegetation or valvular abnormalities were observed, but the pulmonic valve was not well visualized.

**Figure 3:**
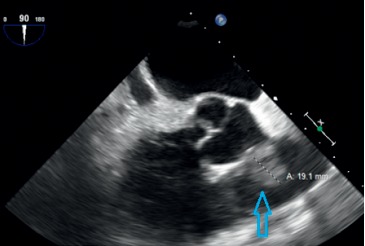
TEE showed large vegetation (blue arrow) over the pulmonic valve.

Blood culture persistently was positive with MRSA, even after the antibiotic was exchanged. Repeated CT scan chest without IV contrast reveals progression in lung pneumonia and increase in size of the existing nodules and cavitations, as well as multiple new nodules. Due to the high concern of cardiovascular system focal infection, another TEE was performed after one week after the first one. At this time, a large mass compatible with vegetation was seen over the pulmonic valve measured ∼1.9 × 0.9  cm (Figure 3).

Due to uncontrolled source of infection, persistent bacteremia, and the size of the vegetation, the patient was evaluated by cardiothoracic surgery team; then, the patient underwent pulmonary valve replacement using a LivaNova extra-large sutureless valve.

**Figure 4:**
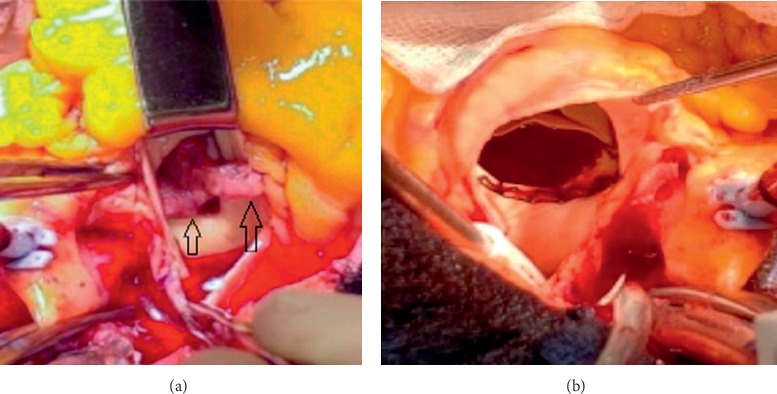
(a) Image from the surgical field showing large vegetations (black arrows) over the valve prior to the resection. (b) Image from surgical field after pulmonic valve resection along with all the vegetations.

Peroperative note: a large vegetation was involving the pulmonic valve, which was adherent to the pulmonary artery itself (Figures 4(a) and 4(b)).

**Figure 5:**
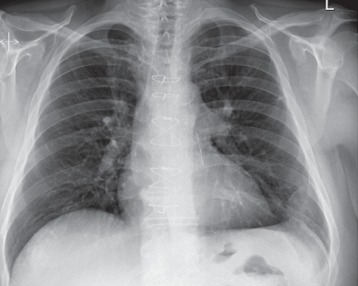
CXR after completion of the therapy with IV vancomycin.

Tissue valve culture came back positive for MRSA, sensitive to vancomycin MIC 1  mg/ml, and resistant to levofloxacin. Repeated blood culture showed sterile blood. The patient was discharged home on IV vancomycin with goal vancomycin trough level 15–20  mg/ml for a total of 6 weeks via the central line. Repeated CXR after completion of the course of therapy showed total resolution of all lung nodules (Figure 5).

## 3. Discussion

Many risk factors can lead to infective endocarditis (IE), including injection drug use, poor dentation, previous history of endocarditis, prosthetic heart valve, bicuspid aortic valve, ventricular septal defect, and hemodialysis patients. The most common pathogens involving in the infection are *staphylococci*, *streptococci*, and *enterococci*, which account up to 50% of all cases of endocarditis. *Staphylococcus aureus* is the leading cause of IE in many regions in the world, and in the United States, methicillin-resistant *Staphylococcus aureus* (MRSA)-IE represent 37.2% of all IE cases [1].

Colonization with MRSA increases the risk of complication including bacteremia, especially in patients with chronic diseases such as diabetes mellitus or immunocompromised patients. Based on national prevalence of MRSA in 2010 in the United States, the overall MRSA prevalence rate was 66.4 per 1000 inpatients (25.3 infections and 41.1 colonizations per 1000 patients) [2]. However, MRSA colonization varied between studies depending on the methodology used. A paper published in “*The Journal of Infectious Diseases*” in 2008 by Gorwitz determines that the risk factors for colonization with MRSA in US females were age ≥60 years, diabetes, and poverty, whereas in US males the only significant risk factor was healthcare exposure [3].

Pulmonic valve endocarditis (PV-IE) is a rare condition, and it accounts around 1.1% of autopsies with valvular endocarditis [4], based on an article published by Cassling in 1985. A similar result was found in another paper released in 2003 in *British Medical Journal* (BMJ) reported by Dhakam and Jafary. Only 38 cases of structurally normal native PV-IE have been reported in the literature, between 1960 and 2000, which account for 1.5–2.0% of all cases of endocarditis [5]. Reviewing literatures from 1960 to 1999, only 36 cases of PV-IE were reported, done by Ramadan and his colleagues, published in *Canadian Journal of Cardiology* [6].

Many theories were used to explain the rarity of PV-IE including low pressure gradient within the right heart, lower oxygen content of venous blood, and differences in the covering and vascularization of the right heart endothelium [6]. However, existing risk factors for right-sided heart endocarditis, such as intravenous drugs abuse, central venous catheter, pacemaker implantation, congenital heart disease, and liver or kidney transplantation [7], are very important to suspect of tricuspid valve endocarditis and/or pulmonic valve infective endocarditis in the presence of multiple lung lesions compatible with septic emboli.

Nine cases of PV-IE were identified in Mayo Clinic between 2000 and 2014: Three of them with congenital heart disease, two with central venous catheters, and three with cardiovascular implantable electronic device [8]. Our patient has no predisposing risk factors from what was mentioned above, but he does have a risk factor for MRSA bacteremia due to the colonization with MRSA.

The usual presentation of the patient with pulmonic valve infective endocarditis is fever, pleuritic chest pain, hemoptysis, cough, and shortness of breath due to pulmonary septic emboli. However, the pulmonic regurgitant murmur is often a late feature, as it was reported by Schroeder in 2005 [9].

The difficulty in diagnosing PV-IE is due to inability to properly visualize the pulmonic valve. Transesophagal echocardiogram (TEE) of PV is challenging due to its position, which is the most anterior and farthest from the TEE probe in comparison to other heart valves. While the TEE failed to detect one case of PV-IE from all nine cases reported in the literature review in Mayo Clinic, the transthoracic echocardiogram (TTE) was diagnostic in all isolated cases [8].

## 4. Conclusion

Pulmonic valve endocarditis is a rare disease and the diagnosis is challenging in absence of risk factors, as it was reviewed in the discussion. Our patient was persistently bacteremic with recurrent septic emboli to the lungs which raised our suspicion of right-sided heart endocarditis, despite inconclusive first TEE report. Therefore, repeated TEE was performed, and it showed large vegetation involving the pulmonic valve. Treatment was according to general endocarditis guidelines.
